# Meteorological Register for December, 1816

**Published:** 1817-03

**Authors:** John Bailey

**Affiliations:** Surgeon, &c.


### METEOROLOGICAL REGISTER for DECEMBER, 1816.

*Kept at Harwich, in Essex.*

By JOHN BAILEY, Surgeon, &c.

| Moon's Age. | | Atmospherical Pressure. Morn. | Ev. | Temp. | Wind. | Remarks. | General Statement of Diseases. |
|---|---|---|---|---|---|---|---|
| | 1 | 30 5 | 30 3½ | 32 | N | Foggy and dull | |
| | 2 | 2½ | 2½ | 35 | W | ditto | |
| | 3 | | | 37 | | ditto | |
| ○ | 4 | | 2 | 39 | NW | ditto | |
| | 5 | 1 | 29 5½ | 41 | SW | Rain | |
| | 6 | 29 5 | 4 | 39 | SW | ditto | |
| | 7 | 4 | 6 | 37 | NW | Fine and clear | |
| | 8 | 5 | 6½ | 39 | W | ditto | |
| | 9 | 6½ | 6½ | 35 | SE | | |
| | 10 | 5 | 2 | 41 | W | Rain | |
| | 11 | 2 | 4 | 40 | NW | ditto | |
| ☽ | 12 | 2 | 28 | 40 | SW | Rain, G. of W. | |
| | 13 | 28 9½ | 2 92 | 37 | W | Fine and clear | |
| | 14 | 29 3 | | 34 | W | Rain G. of W. | |
| | 15 | 28 2 | 29 1½ | 44 | W | ditto | |
| | 16 | 29 3 | 4½ | 33 | W | Fine and clear | |
| | 17 | 2 | 2 | 48 | W | Rain | |
| ● | 18 | 2½ | 7 | 38 | NW | ditto | |
| | 19 | 9 | 30 2 | 35 | N | Snow | |
| | 20 | 30 3½ | 3½ | 33 | N | ditto | |
| | 21 | 2½ | 2½ | 30 | SE | | |
| | 22 | | | 27 | NW | | |
| | 23 | 29 9 | 29 7 | 30 | W | Rain | |
| | 24 | 6 | 5 | 45 | W | Dull | |
| | 25 | 7 | 6 | 38 | NW | Fine and clear | |
| ☾ | 26 | 5 | 4 | 45 | SW | Rain, G. of W. | |
| | 27 | 4½ | 6 | 37 | SW | Fine and clear | |
| | 28 | 7 | 2½ | 33 | SW | Rain, G. of W. | |
| | 29 | 2½ | 8 | 35 | SW | ditto | |
| | 30 | 8 | 6½ | 43 | SW | ditto | |
| | 31 | 7 | 7 | 44 | SW | ditto | |

---

The last column of the present register is unusually barren. It rarely happens, that the month of December, in the crowded and confined population of a town is free from epidemical disease. With the exception of a very few slight catarrhal complaints, there has been no atmospherical disorder either in the town or neighbourhood.

